# High spatial resolution electrochemical biosensing using reflected light microscopy

**DOI:** 10.1038/s41598-019-50949-9

**Published:** 2019-10-23

**Authors:** Raluca-Elena Munteanu, Ran Ye, Cristina Polonschii, Adrian Ruff, Mihaela Gheorghiu, Eugen Gheorghiu, Rabah Boukherroub, Wolfgang Schuhmann, Sorin Melinte, Szilveszter Gáspár

**Affiliations:** 1 International Centre of Biodynamics, Intrarea Portocalelor 1B, 060101 Bucharest, Romania; 2 Institute of Information and Communication Technologies, Electronics and Applied Mathematics, Université catholique de Louvain, 1348 Louvain-la-Neuve, Belgium; 3 Analytical Chemistry - Center for Electrochemical Sciences (CES), Faculty of Chemistry and Biochemistry, Ruhr-University Bochum, Universitätsstr. 150, D-44780 Bochum, Germany; 4 Univ. de Lille, CNRS, Centrale Lille, ISEN, Univ. Valenciennes, UMR 8520-IEMN Lille, France

**Keywords:** Electrochemistry, Analytical chemistry, Bioanalytical chemistry, Sensors, Techniques and instrumentation

## Abstract

If the analyte does not only change the electrochemical but also the optical properties of the electrode/solution interface, the spatial resolution of an electrochemical sensor can be substantially enhanced by combining the electrochemical sensor with optical microscopy. In order to demonstrate this, electrochemical biosensors for the detection of hydrogen peroxide and glucose were developed by drop casting enzyme and redox polymer mixtures onto planar, optically transparent electrodes. These biosensors generate current signals proportional to the analyte concentration via a reaction sequence which ultimately changes the oxidation state of the redox polymer. Images of the interface of these biosensors were acquired using bright field reflected light microscopy (BFRLM). Analysis showed that the intensity of these images is higher when the redox polymer is oxidized than when it is reduced. It also revealed that the time needed for the redox polymer to change oxidation state can be assayed optically and is dependent on the concentration of the analyte. By combining the biosensor for hydrogen peroxide detection with BFRLM, it was possible to determine hydrogen peroxide in concentrations as low as 12.5 µM with a spatial resolution of 12 µm × 12 µm, without the need for the fabrication of microelectrodes of these dimensions.

## Introduction

Electrochemical detection usually combines high sensitivity and short response time with excellent spatial resolution only if the dimensions of the working electrode are reduced using nano- and micro-fabrication methods^1,2^. Unfortunately, almost all electrode miniaturization methods are relatively complex, multistep processes, which are very often also expensive and time consuming. Individually addressing the electrodes of high density microelectrode arrays imposes additional technological challenges. This situation raised the question of whether alternative ways to provide electrochemical detection with spatial resolution are available. Optical microscopy methods can provide the necessary spatial resolution to electrochemical detection given that the involved electrochemical processes do not only change the electrochemical, but also the optical properties of the electrode/solution interface.

Some electrochemical processes induce significant changes of the refractive index of the solution adjacent to the electrode and such changes can be sensitively monitored using Surface Plasmon Resonance (SPR)-based methods. Indeed, SPR imaging and SPR microscopy were already used to provide electrochemical detection with spatial resolution. For example, it was demonstrated that these optical methods can be used to map current heterogeneities characterizing electrodes during the electrochemical oxidation and reduction of hexaammineruthenium^3^ and hexacyanoferrate^4^. Some electrochemical methods (e.g. non-faradaic Electrochemical Impedance Spectroscopy, EIS) do not significantly impact the refractive index of the solution adjacent to the electrode, but instead modulate the surface charge density of the electrode. As this impacts SPR signals, SPR microscopy was also applied to provide spatial resolution to some EIS experiments^5–7^. Such SPR-based developments were recently reviewed elsewhere^8^.

In addition to the SPR-based methods, dark field microscopy and fluorescence microscopy were also integrated with electrochemistry in order to provide spatial resolution to the latter. These combined, opto-electrochemical approaches were used to assess the electrochemistry of single nanoparticles (e.g. conjugated polymer nanoparticles^9^, silver nanoparticles^10,11^, cobalt nanoparticles^12^, etc.) and complete the purely electrochemical observation of single nanoparticle collision events^13^. Instead of measuring the current given by a nanoparticle, the microscopy methods determine the local current generated by a nanoparticle by means of optical signals. These approaches were also recently reviewed elsewhere^14^.

**Figure 1:**
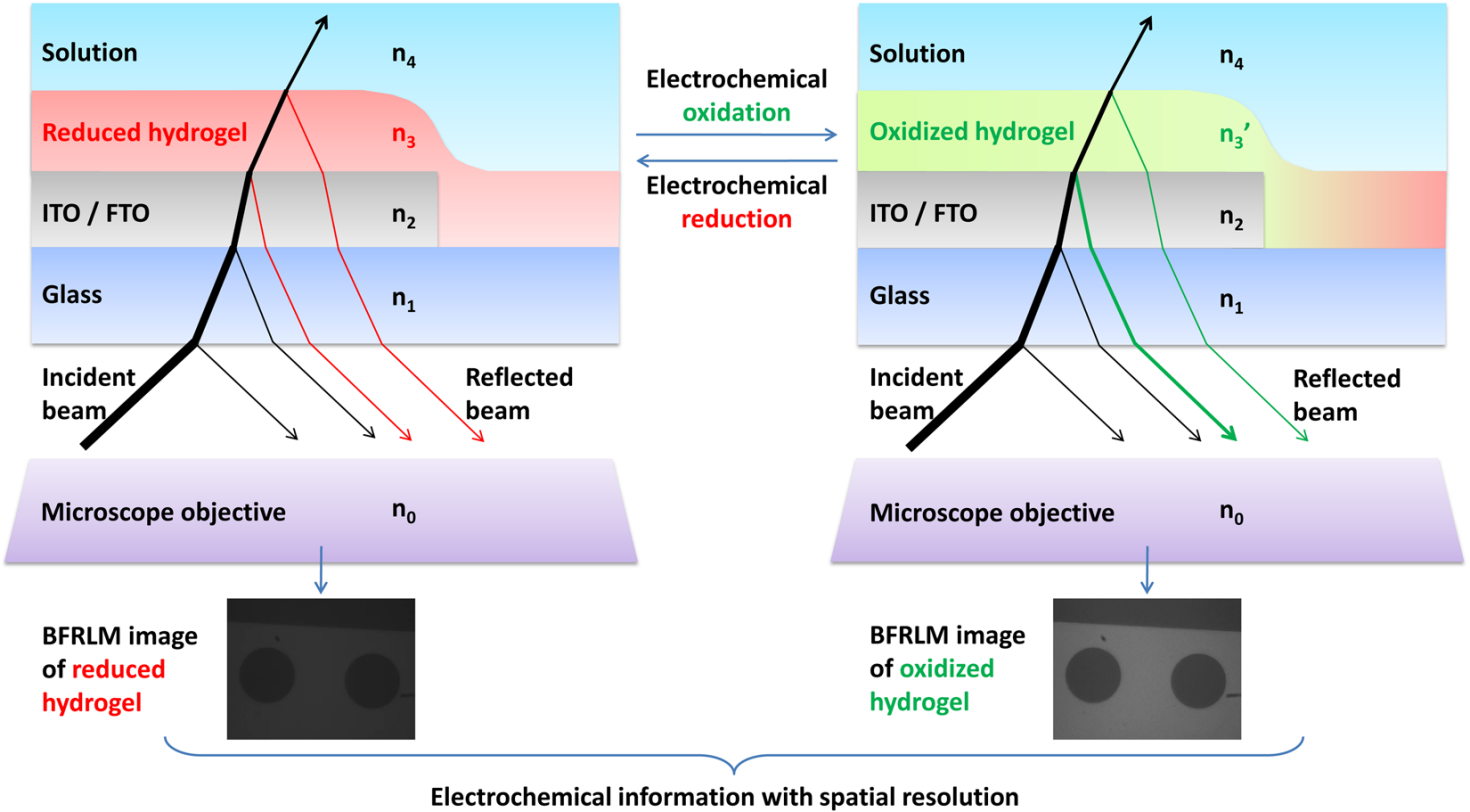
Schematics of BFRLM for increasing the spatial resolution of redox hydrogel-based electrochemical biosensors. The biosensors are obtained by modifying optically transparent metal oxide electrodes fabricated onto planar glass slides with redox hydrogels consisting of cross-linked redox polymers and enzymes. The incident light is refracted and reflected on the different interfaces of the multilayered sensor. The refractive indices of the glass (n_1_), of the metal oxide (n_2_), of the redox hydrogel (n_3_), and of the solution (n_4_) define how much of the incident light is returned and analyzed. Only the refractive index of the hydrogels is changing significantly because of the oxidation or reduction of the redox polymer from their composition. Thus, the BFRLM images recorded during our experiments are essentially high-resolution maps of the oxidation state of the redox hydrogels.

Here we report that the oxidation state of an osmium complex-based redox polymer^15^ can be monitored with bright field reflected light microscopy (BFRLM)^16^. Thus, BFRLM can be used to increase the spatial resolution of redox polymer-based electrochemical biosensors. The proposed approach is schematically depicted in Fig. 1. Compared to the other optical methods used in opto-electrochemical approaches, BFRLM requires a simple illumination scheme: it does not need light at a certain wavelength or light at a defined angle of incidence. Revealing chemical gradients created by metabolically active single cells and mapping the heterogeneity of electrocatalytic interfaces used in biosensing or energy conversion are among the potential applications requiring such spatial resolution.

## Results and Discussion

### Properties of the electrodes used for building biosensors

**Figure 2:**
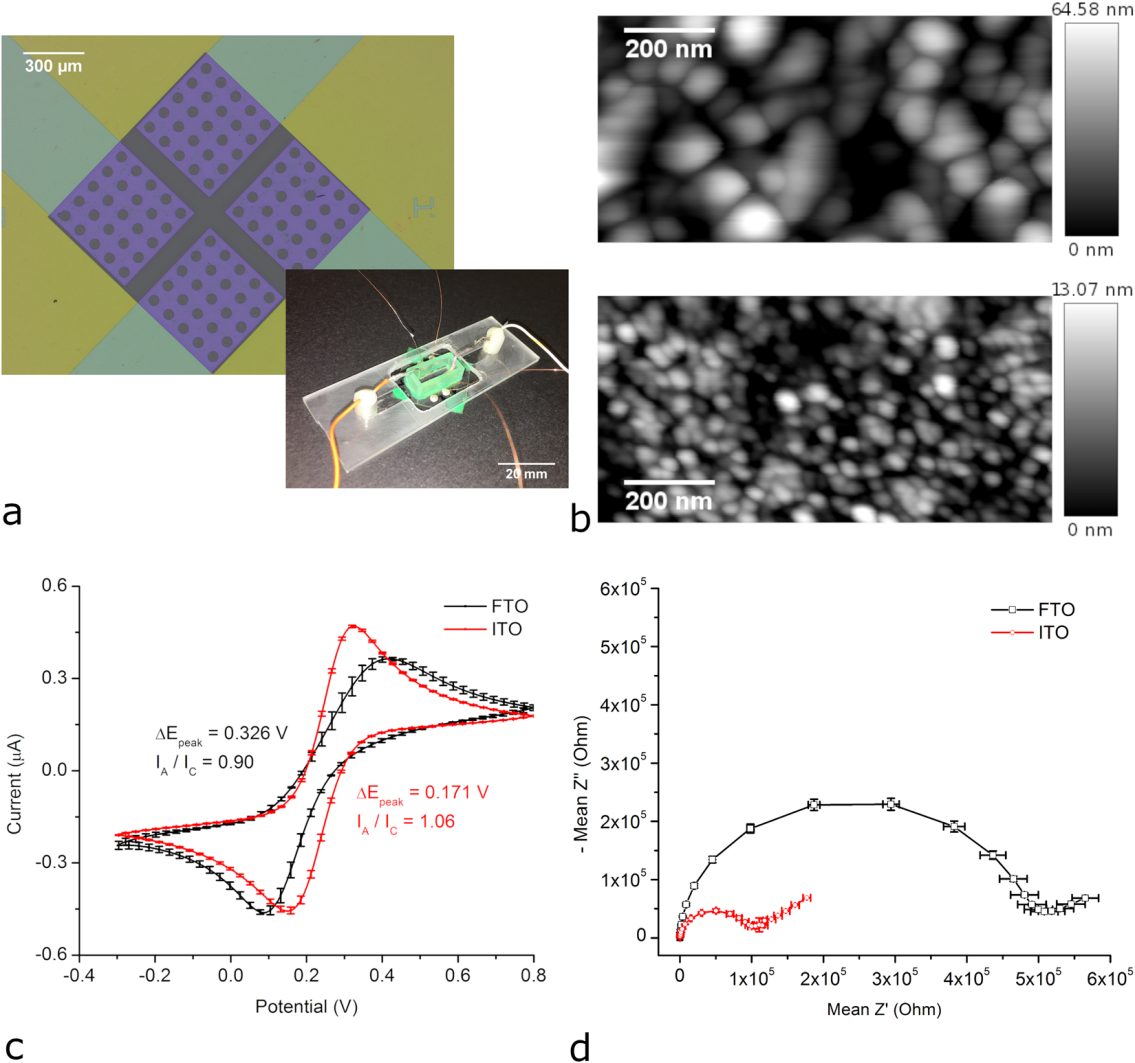
Properties of the electrodes used for building biosensors. (**a**) Images of an ITO electrode array and of the electrochemical cell facilitating the simultaneous optical and electrochemical investigation of such electrode arrays. (**b**) AFM height images recorded on a FTO electrode (upper image) and on an ITO electrode (lower image). (**c**) Cyclic voltammograms recorded at 0.050 V s^−1^ in the presence of 1 mM ferricyanide and 1 mM ferrocyanide in 0.1 M phosphate buffer. (**d**) Nyquist plots of the EIS spectra recorded in the frequency range from 100 kHz to 0.1 Hz in presence of 1 mM ferricyanide and 1 mM ferrocyanide in 0.1 M phosphate buffer pH 7.4.

Electrodes to be used in our approach have to satisfy three requirements. First, they have to be transparent in order to facilitate the observation of the electrode/solution interface with an inverted microscope. Second, they have to be fabricated on ~170 µm thick glass in order to be compatible also with microscope objectives with high magnification and short working distance. Third, they have to expose electrochemically inactive areas (e.g. circular holes) enabling background subtraction to eliminate noise arising from, for example, fluctuations of the incident light. The electrodes do not have to be small as spatial resolution will be provided by BFRLM. Because such electrodes are not commercially available, arrays consisting of four planar, optically transparent, square shaped electrodes made of either fluorine doped tin oxide (FTO) or indium tin oxide (ITO) were fabricated. Figure 2a shows images of one of the ITO electrode arrays and of the electrochemical cell facilitating the simultaneous optical and electrochemical investigation of such electrode arrays (see also Supplementary Information, Figs S1 and S2).

Since the morphological and electrochemical properties of FTO and ITO thin films are sensitive to the fabrication conditions, the fabricated electrodes were investigated using atomic force microscopy (AFM), scanning electron microscopy (SEM), cyclic voltammetry and EIS. Figure 2b shows AFM height images revealing that FTO electrodes are characterized by a larger surface roughness than ITO electrodes (~12 nm *vs*. ~2 nm average roughness, and ~90 nm *vs*. ~20 nm peak-to-valley roughness). The larger surface roughness of FTO electrodes was also confirmed by SEM (Fig. S1c,d). Figure 2c shows cyclic voltammograms recorded with the metal oxide electrodes immersed in a solution containing hexacyanoferrate. These voltammograms were obtained by averaging the voltammograms of the four electrodes located on the same glass substrate. The small error bars (representing standard deviations) indicate the good reproducibility of the electrode fabrication process. Good reproducibility was also observed in between electrodes from different glass substrates. The voltammograms obtained with the ITO electrodes are characterized by smaller peak potential differences (0.171 V vs. 0.326 V for FTO) and by peak current ratios closer to 1 (1.06 vs. 0.90 for FTO), indicating that the ITO electrodes have improved electrochemical properties as compared to the FTO electrodes. Figure 2d depicts the Nyquist plots of EIS spectra recorded using ITO and FTO electrodes immersed in a solution containing hexacyanoferrate. These spectra were obtained by averaging the spectra of the four electrodes on the same glass substrate. They confirm that the ITO electrodes exhibit superior electrochemical properties as compared with the FTO electrodes. The charge transfer resistance of the ITO electrodes is roughly 5 times smaller than that of the FTO electrodes (~100 kΩ vs. ~500 kΩ) when using hexacyanoferrate as redox probe.

### Electrochemical detection of hydrogen peroxide and glucose using FTO and ITO electrodes modified with redox hydrogels

**Figure 3:**
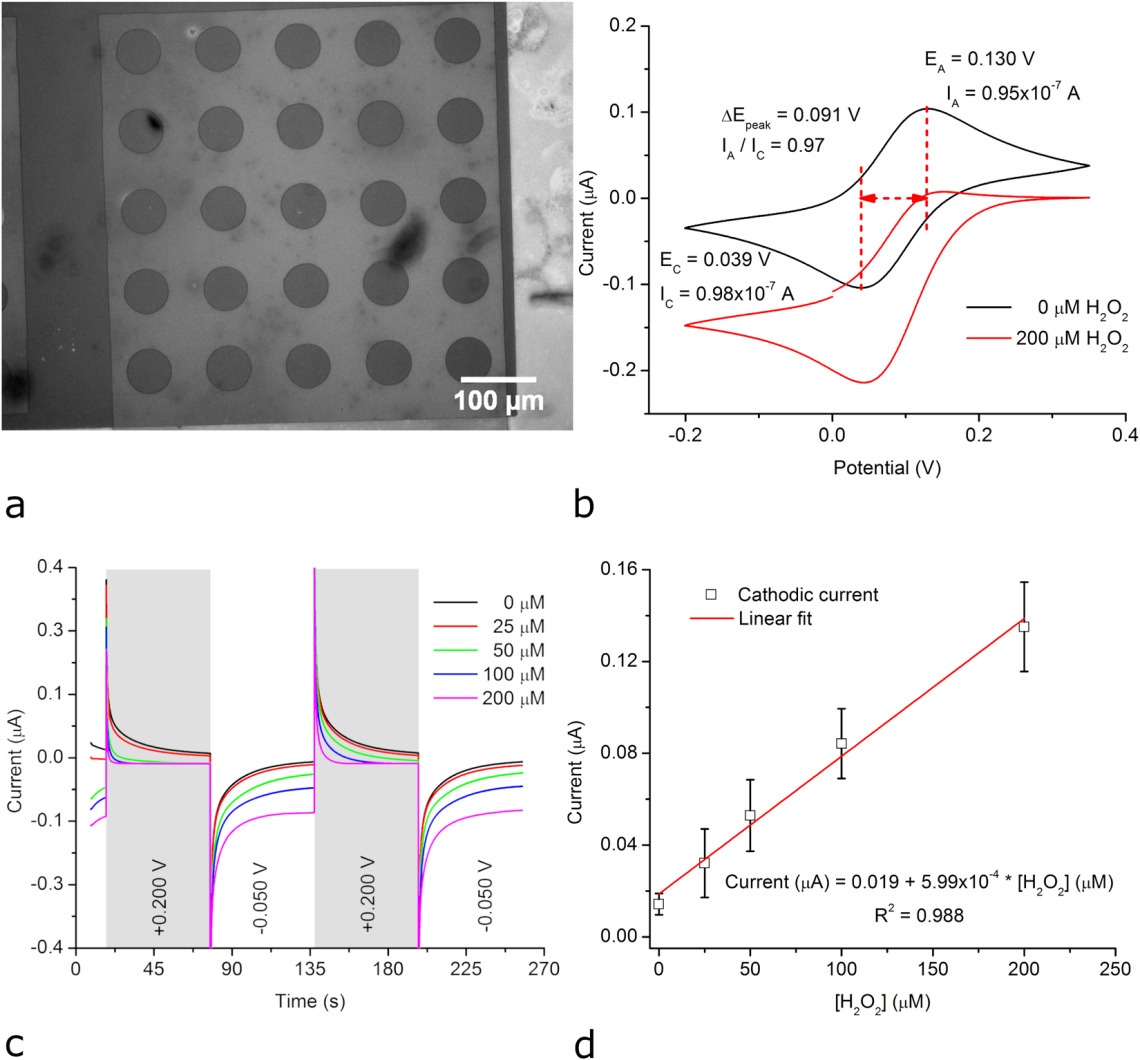
Electrochemical detection of hydrogen peroxide using a FTO electrode modified with HRP-based redox hydrogel. (**a**) BFRLM image of the FTO electrode. (**b**) Cyclic voltammograms of the modified FTO electrode recorded at a scan rate of 0.010 Vs^−1^ in the absence and presence of hydrogen peroxide. (**c**) Current signal of the modified FTO electrode polarized to potentials oxidizing/reducing the redox polymer while the hydrogen peroxide concentration was stepwise increased. (**d**) Calibration curve derived from the current values recorded at the end of the second cathodic pulse (at *t* = 256 s).

FTO electrodes, such as the one shown in Fig. 3a, were modified with redox hydrogels consisting of horseradish peroxidase (HRP), an osmium complex-based redox polymer, and poly(ethylene glycol) diglycidyl ether (PEGDGE) cross-linker (see Methods section). As shown in Fig. 3b, these hydrogel-modified FTO electrodes exhibit cyclic voltammograms with two current peaks corresponding to the oxidation and the reduction of the redox polymer (at +0.130 V and +0.039 V, respectively). The observed peak potential separation (0.091 V) and peak current ratio (0.97) are specific for a quasi-reversible redox couple. Moreover, in the presence of hydrogen peroxide, the current corresponding to the reduction of the redox polymer increased and the current corresponding to the oxidation of the redox polymer decreased, as expected for an electrochemical process coupled to an enzyme-catalysed reaction^17^.

We also carried out chronoamperometric experiments in which the potential of the hydrogel-modified electrodes was set to either +0.200 V (to oxidize the redox polymer) or to −0.050 V (to reduce the redox polymer) and the concentration of the hydrogen peroxide in the solution was increased stepwise from 0 to 200 µM. The cathodic currents increased and the anodic currents decreased with increasing hydrogen peroxide concentrations in these experiments as well (Fig. 3c). Figure 3d displays a calibration curve derived from the cathodic current recorded at the end of the second cathodic pulse (i.e., at *t* = 256 s). This calibration curve (average of 5 experiments with 5 different hydrogel-modified FTO electrodes) is linear over the investigated hydrogen peroxide concentration range (i.e., up to 200 µM). The coefficient of variation characterizing the sensitivity (i.e., the slope of the calibration curve shown in Fig. 3d) was 15%. The detection limit, calculated as the hydrogen peroxide concentration giving a signal equal to the blank plus three standard deviations of the blank, was found to be 15 µM. The limit of quantitation, calculated as the hydrogen peroxide concentration giving a signal equal to the blank plus ten standard deviations of the blank, was found to be 70 µM. These results confirm that FTO electrodes modified with such HRP-based redox hydrogels are able to generate a current signal proportional to the hydrogen peroxide concentration via a reaction sequence in which HRP catalytically converts hydrogen peroxide and ultimately oxidizes the redox polymer. This reaction sequence is detailed elsewhere^18^ and also depicted in Fig. S3a. Similar electrochemical results were obtained when ITO electrodes were modified with HRP-based redox hydrogels (Fig. S4).

FTO and ITO electrodes were also modified with redox hydrogels containing glucose oxidase (GOx) instead of HRP and then investigated using cyclic voltammetry and chronoamperometry in presence of varying concentrations of glucose. It was observed that, the higher the concentration of glucose in the solution, the faster the redox polymer is reduced, and the larger the oxidation currents in both cyclic voltammetry and chronoamperometry are (Figs S5 and S6). These results confirm that FTO and ITO electrodes modified with such GOx-based redox hydrogels are able to generate a current signal proportional to the glucose concentration via a reaction sequence in which GOx catalytically converts glucose and ultimately reduces the redox polymer. This reaction sequence is detailed elsewhere^19^ and also depicted in Fig. S3b.

### Opto-electrochemical detection of hydrogen peroxide using FTO and ITO electrodes modified with HRP-based redox hydrogel

**Figure 4:**
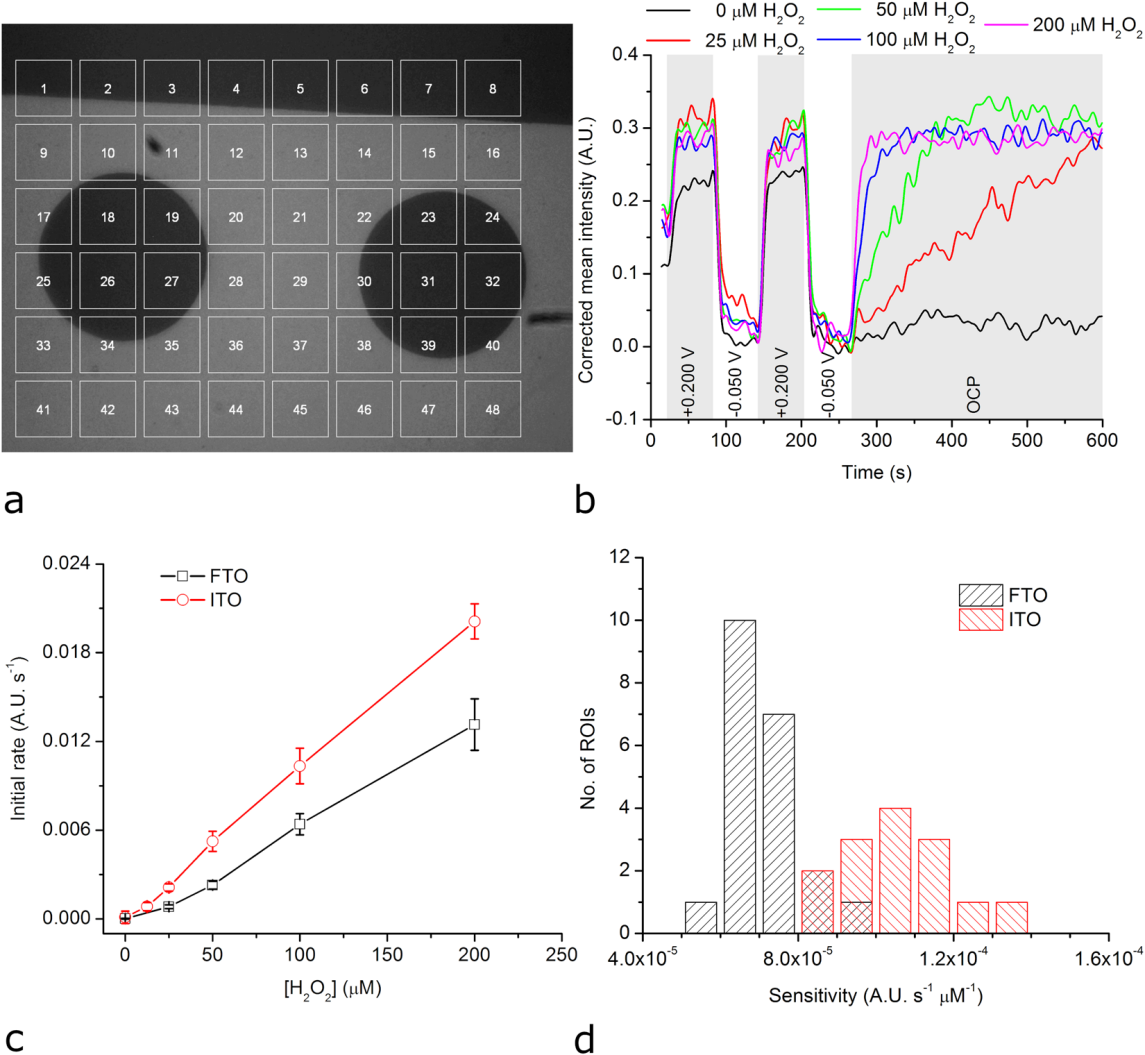
Opto-electrochemical detection of hydrogen peroxide using FTO or ITO electrodes modified with HRP-based redox hydrogel. (**a**) BFRLM image with 48 ROIs (with dimensions of 18 µm × 18 µm) of a FTO electrode modified with HRP-based redox hydrogel. (**b**) Evolution of the optical signal for ROI no. 28 when the FTO electrode modified with HRP-based redox hydrogel was first polarized to potentials oxidizing/reducing the redox polymer and then kept at OCP while the hydrogen peroxide concentration was increased stepwise. (**c**) Average calibration curves obtained by quantifying the optically observed initial rate of the biocatalytic reaction in the presence of different hydrogen peroxide concentrations. (**d**) Sensitivity distribution of the different ROIs.

Chronoamperometric experiments, such as those described above, were made while simultaneously BFRLM images of the electrode/solution interface were recorded through the 40 × magnification objective of an inverted microscope. Figure 4a shows a BFRLM image with 48 regions of interest (ROIs) of a FTO electrode modified with HRP-based redox hydrogel. This BFRLM image (the first out of 600 time lapse images) highlights areas with conductive metal oxide (and high intensity) as well as areas with nonconductive glass (and low intensity). Figure 4b reveals how the mean intensity of one of the ROIs (found on FTO) changes when the potential of the electrode is pulsed twice to + 0.200 V (to oxidize the redox polymer), twice to −0.050 V (to reduce the redox polymer), and then left at open circuit potential (OCP; starting with *t* = 257 s) both in the absence and in the presence of different concentrations of hydrogen peroxide. As one can observe, the mean intensity of the ROI increases when the redox polymer is oxidized and decreases when the redox polymer is reduced. More importantly, when the electrode modified with the HRP-based hydrogel is kept at OCP (starting with *t* = 257 s), the mean intensity of the ROI increases towards the level of the fully oxidized redox polymer with a slope that depends on the hydrogen peroxide concentration. The higher the hydrogen peroxide concentration is, the greater this slope becomes. Hence, the analysis of the BFRLM images confirms that i.) the reduced form and the oxidized form of the redox polymer exhibit different optical properties, and ii.) the time needed to biocatalytically oxidize the redox polymer after it was electrochemically reduced depends on the concentration of hydrogen peroxide. For a thorough understanding of our approach, let us analyse the physical origins of the optical signal shown in Fig. 4b. When light hits the metal oxide-redox hydrogel interface, part of it is reflected back to the objective and part of it is refracted. The refracted light travels through the redox hydrogel, it is absorbed by the redox hydrogel (as this is colored) and then again reflected and refracted on the redox hydrogel-solution interface; thus, the refractive indices (and the absorption properties) of the glass, of the metal oxide, of the redox hydrogel, and of the solution will define how much of the incident light is returned, captured by the CCD of the camera, and then analyzed (see also Fig. 1). However, during our experiments only the refractive index and the absorption of the redox hydrogel are changing significantly through the oxidation and reduction of the redox polymer from its composition. Moreover, the oxidation and the reduction of the redox polymer can advantageously be induced both electrochemically (see signal at *t* < 257 s) and biochemically (see signal at *t* > 257 s), using the substrate of the enzyme found in the hydrogel (Fig. S3). Figure 4c shows the average calibration curve reflecting the sensitivity to hydrogen peroxide of the FTO electrode modified with the HRP-based hydrogel. This curve was obtained by (i) determining the initial rate of the biocatalytic reaction at different hydrogen peroxide concentrations (as the slope of the mean intensity increase recorded after *t* = 257 s, when the electrode is left at OCP, see Fig. 4b), and (ii) averaging the initial rates calculated from all ROIs found on FTO for a given hydrogen peroxide concentration. In terms of linear range, this calibration curve resembles the calibration curve obtained when using purely electrochemical readout (see Fig. 3d *vs*. Fig. 4c). The average sensitivity of the ROIs, calculated as the slope of the average calibration curve, was 6.8 × 10^−5^ A.U. s^−1^ µM^−1^. The coefficient of variation characterizing the average sensitivity was 14%. The detection limit and the limit of quantitation were found to be 15 µM and 20 µM, respectively. While no ROI on nonconductive glass was found suitable for hydrogen peroxide sensing, all ROIs on the FTO electrode were found to act as tiny, functional hydrogen peroxide sensors characterized by a relatively narrow sensitivity distribution (see Fig. 4d). Evidently, BFRLM can increase the spatial resolution of hydrogen peroxide electrochemical sensors made using FTO electrodes and HRP-based redox hydrogels. Our ROIs have dimensions of 100 pixels × 100 pixels and, thus, are equivalent of microelectrodes as small as 18 µm × 18 µm. However, unlike microfabricated microelectrodes, they are easily reconfigurable (in terms of size and shape) and do not require electrode leads or contact pads. Therefore, they can be more densely packed than microelectrode arrays.

Next, we investigated the differences between ITO and FTO electrodes in BFRLM. Moreover, we aimed to gain additional spatial resolution by switching to a higher magnification objective. The results obtained with the ITO electrode and a 63 × magnification objective are qualitatively similar to those achieved with the FTO electrode and the 40 × magnification objective (Fig. S7). However, the evolution of the corrected mean intensity while changing the applied potentials and the hydrogen peroxide concentration is somewhat noisier. The 48 ROIs, although still 100 pixels × 100 pixels each, correspond now to microelectrodes with dimensions of 12 µm × 12 µm, that is, to microelectrodes with about ~ 55% smaller surface area than the ones discussed above. This decrease of the dimensions of the ROIs explains the noisier optical signals. The sensitivity of the ROIs to hydrogen peroxide was nevertheless preserved (Fig. 4c). The average sensitivity of the ROIs was 1.02 × 10^−4^ A.U. s^−1^ µM^−1^ with coefficient of variation of 13%. Compared to the average sensitivity of the ROIs on the FTO-based biosensor, the average sensitivity of the ROIs on the ITO-based biosensor is roughly 33% higher. Interestingly, the sensitivity of the ITO-based biosensor was about 29% higher than that of the FTO-based biosensor also when used with chronoamperometry that interrogates the entire surface of the electrode (see calibration curve obtained using FTO in Fig. 3d and using ITO in Fig. S4c). Therefore, the higher sensitivity of the hydrogen peroxide biosensors on ITO as compared to those on FTO is most probably due to the higher electron transfer rates which characterize the ITO electrodes. These higher electron transfer rates are implied by the smaller peak potential differences and the larger current peaks observed in cyclic voltammetry as well as the smaller charge transfer resistances observed in EIS when using ITO (see Fig. 2c,d). However, contributions from the different optical properties of the two materials cannot be excluded. As with FTO, all ROIs on the ITO electrode were found to act as tiny, functional hydrogen peroxide sensors characterized by a relatively narrow sensitivity distribution (Fig. 4d). The detection limit and the limit of quantitation characterizing the opto-electrochemical hydrogen peroxide sensor built using ITO were 20 µM and 50 µM, respectively.

### Opto-electrochemical detection of glucose using FTO and ITO electrodes modified with GOx-based redox hydrogel

**Figure 5:**
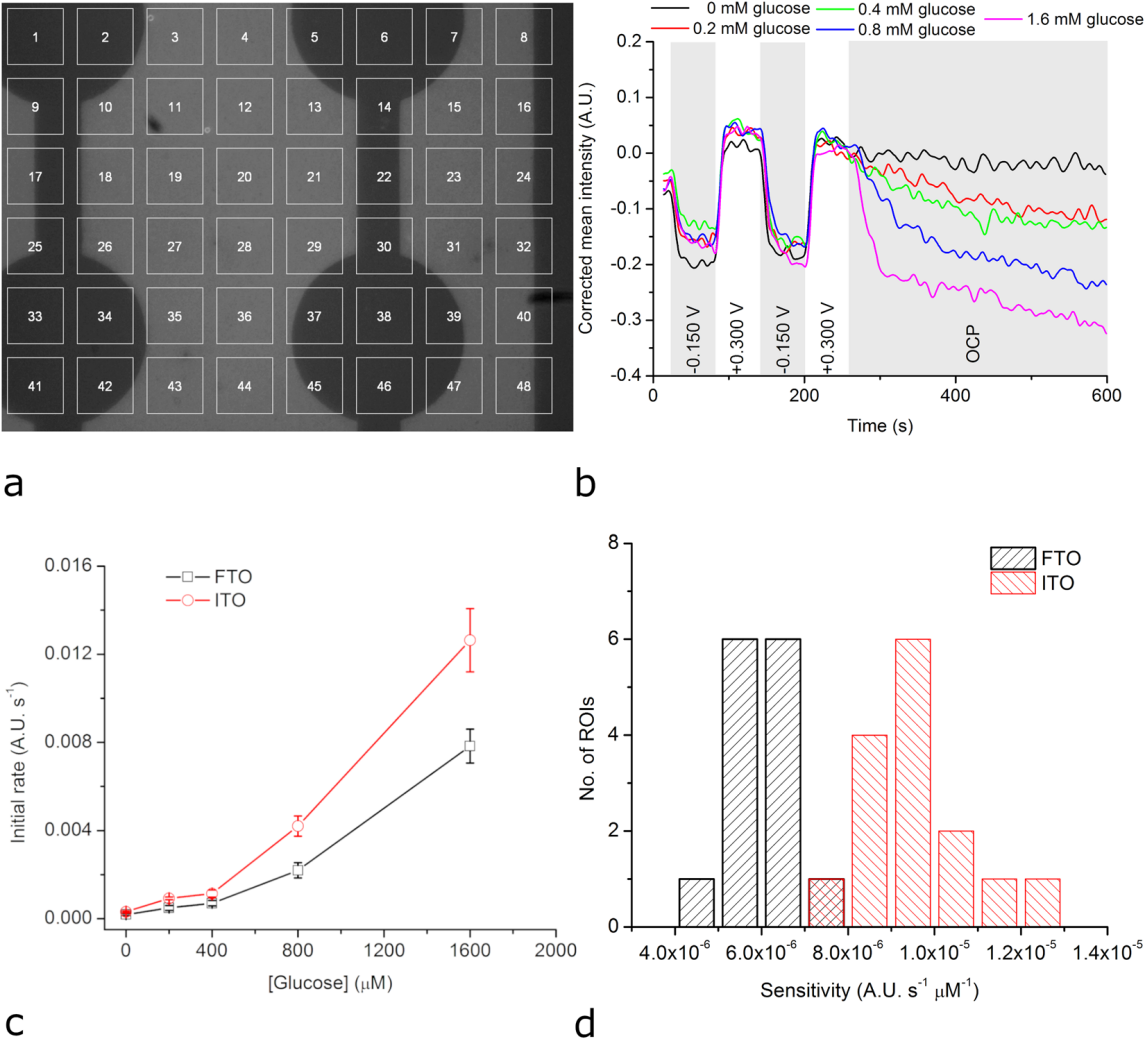
Opto-electrochemical detection of glucose using FTO or ITO electrodes modified with GOx-based redox hydrogel. (**a**) BFRLM image with 48 ROIs with dimensions of 18 µm × 18 µm each of a FTO electrode modified with GOx-based redox hydrogel. (**b**) Evolution of the optical signal for ROI no. 43 when the FTO electrode modified with GOx-based redox hydrogel was first polarized to potentials oxidizing/reducing the redox polymer and then kept at OCP while the glucose concentration was increased stepwise. (**c**) Calibration curves obtained by quantifying the optically observed initial rate of the biocatalytic reaction in the presence of different glucose concentrations. (**d**) Sensitivity distribution of the different ROIs.

Figure 5a shows a BFRLM image of a FTO electrode modified with GOx-based redox hydrogel. Similar images were recorded while the oxidation status of the redox polymer was electrochemically changed both in the absence and in the presence of increasing concentrations of glucose. The GOx-catalyzed oxidation of glucose leads to a reduction of the redox polymer (see Fig. S3b). Therefore, the potential pulse sequence was changed compared to the experiments using HRP in the way that the redox polymer is fully oxidized before the electrode is left at the OCP. When the electrode was left at OCP (i.e., after *t* = 257 s), the mean intensity of the ROI significantly decreased only if glucose was present in solution (Fig. 5b). The higher the glucose concentration is the faster the mean intensity decreases. Intriguingly, the optical signals recorded in the presence of 0.2 to 0.8 mM glucose do not return to the level corresponding to the fully reduced redox polymer even at longer times, possibly due to a competition between the redox polymer and oxygen (the natural co-substrate of GOx). The slope of the initial variation of the mean intensity corresponds to the initial rate of the GOx-catalyzed glucose oxidation and can be used to derive calibration curves reflecting the sensitivity to glucose of each ROI. Figure 5c shows the average calibration curve derived using data from all ROIs found on FTO and reflecting biphasic concentration dependencies. At concentrations up to 0.4 mM glucose, the change of the optical signal with the glucose concentration is smaller than at higher glucose concentrations (from 0.4 to 1.6 mM). Interestingly, the chronoamperometric signal (obtained using the entire FTO electrode) had a very similar behaviour (Fig. 5c *vs*. Fig. S5c). The mean sensitivity of the ROIs (calculated using the linear range of the calibration curves) was 6.1 × 10^−6^ A.U. s^−1^ µM^−1^ with a coefficient of variation of 11%. The sensitivity of the opto-electrochemical glucose biosensor on FTO is roughly one order of magnitude smaller than that of the opto-electrochemical hydrogen peroxide biosensor on FTO. The detection limit and the limit of quantitation characterizing the opto-electrochemical glucose sensor built using FTO were 340 µM and 400 µM, respectively. All ROIs on the FTO electrode were found to act as tiny, functional glucose sensors characterized by a relatively narrow sensitivity distribution (Fig. 5d).

Next, we investigated if ITO electrodes can provide increased sensitivity to glucose biosensors similar to the hydrogen peroxide biosensors. Figure 5c depicts the average calibration curve obtained by combining BFRLM with an ITO electrode modified with GOx-based redox hydrogel (in comparison with the average calibration curve obtained in similar conditions but using a FTO electrode). The average sensitivity of the ROIs was 9.7 × 10^−6^ A.U. s^−1^ µM^−1^ and the coefficient of variation characterizing this sensitivity was 13%. The average sensitivity of the ROIs on the ITO sensor is about 37% higher than that on the FTO sensor. The ITO-based glucose biosensors were also more sensitive to glucose than the FTO-based glucose biosensors for the electrochemical readout (see Supplementary Information, Figs S5 and S6). The detection limit and the limit of quantitation characterizing the opto-electrochemical glucose sensor built using ITO were 320 µM and 350 µM, respectively. Regarding the sensitivity distribution of ROIs defined on ITO, this is somewhat wider than the sensitivity distribution of ROIs defined on FTO (Fig. 5d). Notable, this wider distribution of ROI sensitivities on ITO than on FTO holds also in case of hydrogen peroxide detection (Fig. 4d), suggesting a larger heterogeneity as an intrinsic feature of the ITO electrodes.

## Conclusions

We demonstrate that BFRLM improves the spatial resolution of electrochemical biosensors based on planar, optically transparent electrodes, enzymes, and a redox polymer. The developed opto-electrochemical method exploits the fact that the oxidized and the reduced forms of the redox polymer have different optical properties (i.e. different refractive indices and different absorption coefficients) which can be assessed using an inverted microscope. The feasibility of the proposed method was demonstrated with FTO and ITO electrodes as well as redox hydrogels containing either HRP or GOx. Detection of hydrogen peroxide in concentrations as low as 12.5 µM and with a spatial resolution of 12 µm × 12 µm and of glucose at a concentration as low as 200 µM with a spatial resolution of 18 µm × 18 µm was possible using BFRLM. The figures of merit of our biosensors are compared to the analytical performances of previously described redox hydrogel-based microbiosensors in Table 1. As one can observe, the detection limits of our opto-electrochemical biosensors are somewhat inferior to those of previously described redox hydrogel-based microbiosensors (while other parameters are difficult to compare). However, these figures of merit might be further improved by using redox hydrogels with compositions optimized for achieving the best optical signal, instead of the best electrochemical signal. On the one hand, average sensitivities achieved using ITO electrodes were found to be slightly higher (33–37%) than those obtained with FTO electrodes. On the other hand, ROIs defined on FTO electrodes were characterized by narrower distributions of their sensitivities to hydrogen peroxide and glucose. The developed opto-electrochemical method to detect hydrogen peroxide and glucose uses easily reconfigurable ROIs, which unlike classic microelectrodes require no circuitry and thus can be more densely packed than common microelectrode arrays. The method can be extended to the detection of other analytes which are converted by available enzymes able to accept redox polymers as co-substrate (e.g. glutamate) as well as to multiplexed detection schemes.

**Table 1:** Redox hydrogel-based electrochemical microbiosensors. S = sensitivity; DL = detection limit; LR = linear range; RT = Response time.

Analyte	Electrode	Analytical performances	Array characteristics	Ref.
Hydrogen peroxide	7 µm disk	S = 1.7 µA cm^−2^ µM^−1^; DL = 0.15 µM; LR = 0.5–100 µM; RT = 30 s;	Single site detection; not reconfigurable;	^20^
	10 µm × 400 µm cylinder	S = 7.1 ± 3.2 pA µM^−1^; DL = 285 ± 60 nM; LR = up to 10 µM;	Single site detection; not reconfigurable;	^21^
	10 µm × 300 µm cylinder	S = 111.0 ± 44.9 pA µM^−1^;	Single site detection; not reconfigurable;	^22^
	Array of 48 electrodes 12 µm × 12 µm each	S = 6.8 × 10^−5^ A.U. s^−1^ µM^−1^(FTO); S = 1.02 × 10^−4^ A.U. s^−1^ µM^−1^(ITO); DL = 15 µM (FTO); DL = 20 µM (ITO);	Multisite detection; easily reconfigurable	Present work
Glucose	7 µm disk	S = 20 mA cm^−2^ M^−1^; LR = up to 6 mM; RT = 5 s;	Single site detection; not reconfigurable;	^23^
	0.25 mm disk	S = 0.2 – 0.3 nA mM^−1^; LR = 0–15 mM;	Single site detection; not reconfigurable;	^24^
	0.29 mm disk	S = 1–2.5 nA mM^−1^; LR = up to 60 mM; RT = 60 s;	Single site detection; not reconfigurable;	^25^
	Array of 48 electrodes 18 µm × 18 µm each	S = 6.1 × 10^–6^ A.U. s^−1^ µM^−1^(FTO); S = 9.7 × 10^−6^ A.U. s^−1^ µM^−1^(ITO); DL = 340 µM (FTO); DL = 320 µM (ITO);	Multisite detection; easily reconfigurable;	Present work
Glutamate	5 µm × 300 µm cylinder	S = 3.4 ± 0.94 pA µM^−1^; DL = 1 ± 3 µM; RT = 20–40 s;	Single site detection; not reconfigurable;	^26^
	100 µm disk	S = 0.038 ± 0.005 mA M^−1^; DL = 0.5 µM; LR = up to 50 µM; RT = 35 s;	Single site detection; not reconfigurable;	^27^
	10 µm × 300 µm cylinder	S = 8.5 ± 1.7 pA µM^−1^; DL = 0.09 ± 0.006 µM; RT = 21.9 ± 2.1 s;	Single site detection; not reconfigurable;	^28^

## Methods

### Materials

HRP (Cat. No. P8375), GOx (Cat. No. G7141), Na_2_HPO_4_ (99%), KH_2_PO_4_ (99%), NaCl (99.8%), ferrocyanide (98.5%), hydrogen peroxide (30%), D-(+)-glucose (99.5%) and PEGDGE were all purchased from Sigma Aldrich. Acetone (99.92%) and ethanol (96%) were purchased from Chimreactiv. NaOH (98%) was obtained from Lach-Ner while ferricyanide (99%) was purchased from Merck. All materials were used as received. The osmium complex-based redox polymer was synthesized as described elsewhere^15^. Its redox potential was estimated to be ~+0.190 V *vs*. an Ag/AgCl, 3 M KCl reference electrode. The polymer was used as aqueous stock solution with a concentration of 4.4 mg mL^−1^. Solutions were prepared using ultrapure water from a Direct-Q 3 UV water purification system (from Millipore).

### Fabrication and characterization of planar FTO and ITO electrodes

Square-shaped electrodes (500 µm × 500 µm each) were fabricated on glass slides (2 cm × 2 cm × 170 µm) as detailed in the Supplementary Information (Fig. S1a). Prior to use, all electrodes were cleaned by washing with acetone, ethanol, ultrapure water, 10% NaOH aqueous solution at 80 °C (for 15 min), and again ultrapure water. They were studied with AFM (using a NanoWizard II from JPK Instruments) as well as by cyclic voltammetry and EIS carried out using a PGSTAT128N potentiostat (from Metrohm Autolab).

### Preparation of the redox polymer-based biosensors

For hydrogen peroxide detection, the electrodes were modified by drop casting 5 µL of a mixture of 1 µL of 10 mg mL^−1^ HRP solution, 7 µL of 4.4 mg mL^−1^ redox polymer solution, 2.4 µL of 0.5% (v/v) PEGDGE bifunctional crosslinker, and 1 µL of 0.1 M phosphate buffer solution (pH = 10). Modified electrodes were dried for 1 h at room temperature and overnight at 4 °C. In order to make the electrodes sensitive to glucose, the electrodes were modified using the same procedure in which the 10 mg mL^−1^ HRP solution was replaced with a 10 mg mL^−1^ GOx solution.

### Monitoring the oxidation state of the redox polymer using BFRLM

The redox hydrogel-modified electrodes were integrated into a specifically designed electrochemical cell comprising, in addition to the planar, optically transparent electrodes, a Pt wire counter electrode and an Ag/AgCl wire as quasi-reference electrode (see Supplementary Information; Fig. S2). Compatibility with common microscope stages and unhindered access of the objectives of an inverted microscope, including objectives with high magnifications and very short working distances, to the electrode/solution interface are guaranteed. In a typical opto-electrochemical experiment, the electrochemical cell was filled with 400 µL of phosphate buffer containing 0.1 M Na_2_HPO_4_, 0.1 M KH_2_PO_4_, and 0.01 M NaCl and adjusted to pH 7.4 using NaOH. Then, it was placed onto the stage of an inverted microscope (Observer D1; Carl Zeiss) that was equipped with two objectives (40× and 63×, both Carl Zeiss), a HBO 100 illuminator (Carl Zeiss), and a DFK 31AF03 CCD camera (from The Imaging Source Europe). BFRLM was subsequently used to acquire images of the electrode/solution interface at an acquisition rate of 1 frame per second with an exposure of ~200 ms while the potential at the electrode was set twice to a potential that oxidizes the redox polymer, and twice to a potential which reduces the redox polymer, and then left at the OCP. Images were acquired in this way first in the absence and then in the presence of increasing concentrations of the analyte of interest. Every single opto-electrochemical experiment resulted in a sequence of 600 images revealing how the optical properties of the electrode/solution interface are changed both with respect to the applied potential and the analyte of interest. The opto-electrochemical experiments were repeated at least three times for each analyte and electrode material combination. The analysis of the images obtained in these experiments involved six steps: (1) defining 48 regions of interest (ROIs; 100 pixels × 100 pixels each); (2) measuring the mean intensity of each ROI in each of the 600 images; (3) subtracting the mean intensities of an electrochemically inactive ROI (i.e. a ROI on glass instead on the metal oxide) from the mean intensities of all electrochemically active ROIs in order to eliminate noise (e.g. due to fluctuations of the incident light); (4) plotting the evolution of the corrected mean intensity of each ROI as a function of time; (5) applying a single point baseline offset correction to these plots in order to eliminate small differences in the mean intensities of the same ROI recorded in the presence of increasing concentrations of the analyte of interest; and (6) determining the slope of the linear increase / decrease of the corrected mean intensity that was observed immediately after setting the potential of the electrode to OCP (i.e. after *t* = 257 s). This slope reflects the initial rate of the enzyme catalyzed reaction and is our analyte concentration-proportional analytical signal. Steps 1 and 2 were realized using ImageJ^29^ while the other steps were carried out using various scientific graphing and data analysis software packages.

## Supplementary information

Supplementary Information
