# Supplementary material for: High spatial resolution electrochemical biosensing using reflected light microscopy
Source: Sci Rep. 2019 Oct 23;9:15196. doi: 10.1038/s41598-019-50949-9 (PMC6811617; doi:10.1038/s41598-019-50949-9)
Supplement: Supplementary file 1 — Supplementary Information [file 41598_2019_50949_MOESM1_ESM.pdf]

Supplementary information

High spatial resolution electrochemical biosensing using reflected light microscopy

Raluca-Elena Munteanu[1], Ran Ye[2], Cristina Polonschii[1], Adrian Ruff[3], Mihaela Gheorghiu[1], Eugen Gheorghiu[1], Rabah Boukherroub[4], Wolfgang Schuhmann[3], Sorin Melinte[2], Szilveszter Gáspár[1]*

[1]International Centre of Biodynamics, Intrarea Portocalelor 1B, 060101 – Bucharest, Romania

[2]Institute of Information and Communication Technologies, Electronics and Applied Mathematics, Université catholique de Louvain, 1348, Louvain-la-Neuve, Belgium

[3]Analytical Chemistry - Center for Electrochemical Sciences (CES), Faculty of Chemistry and Biochemistry, Ruhr-University Bochum, Universitätsstr. 150, D-44780 Bochum, Germany

[4] Univ. Lille, CNRS, Centrale Lille, ISEN, Univ. Valenciennes, UMR 8520-IEMN, Lille, France

***Corresponding author:** sgaspar@biodyn.ro

Content

	Page
1. Details on the FTO and ITO electrodes……………………………………..............	**3**
2. Electrochemical cell for opto-electrochemical measurements………………………	**4**
3. Reaction cascades providing the analytical useful signals…………………………..	**5**
4. Electrochemical detection of hydrogen peroxide using an ITO electrode modified with HRP-based redox hydrogel ………..……………………………………………	**5**
5. Electrochemical detection of glucose using a FTO electrode modified with GOx-based redox hydrogel ………………………………………………………………...	**7**
6. Electrochemical detection of glucose using an ITO electrode modified with GOx-based redox hydrogel ………………………………………………………………...	**9**
7. Opto-electrochemical detection of hydrogen peroxide using an ITO electrode modified with HRP-based redox hydrogel……………………………………………...	**11**
8. Opto-electrochemical detection of glucose using an ITO electrode modified with GOx-based redox hydrogel……………………………………………………………..	**12**
9. Selectivity of the developed opto-electrochemical sensors....………………………..	**13**
10. References………………...………………………………………………………...	**15**

1. Details on the FTO and ITO electrodes

The planar, optically transparent FTO and ITO electrodes used throughout the present work were fabricated starting from metal oxide-coated, 3 inch diameter, glass wafers and using the following sequence of six steps: 1.) ITO / FTO deposition, 2.) photolithography, 3.) wet etching of transparent oxide, 4.) silicon nitride deposition, 5.) photolithography and 6.) silicon nitride dry etching (**Fig. S1a**).

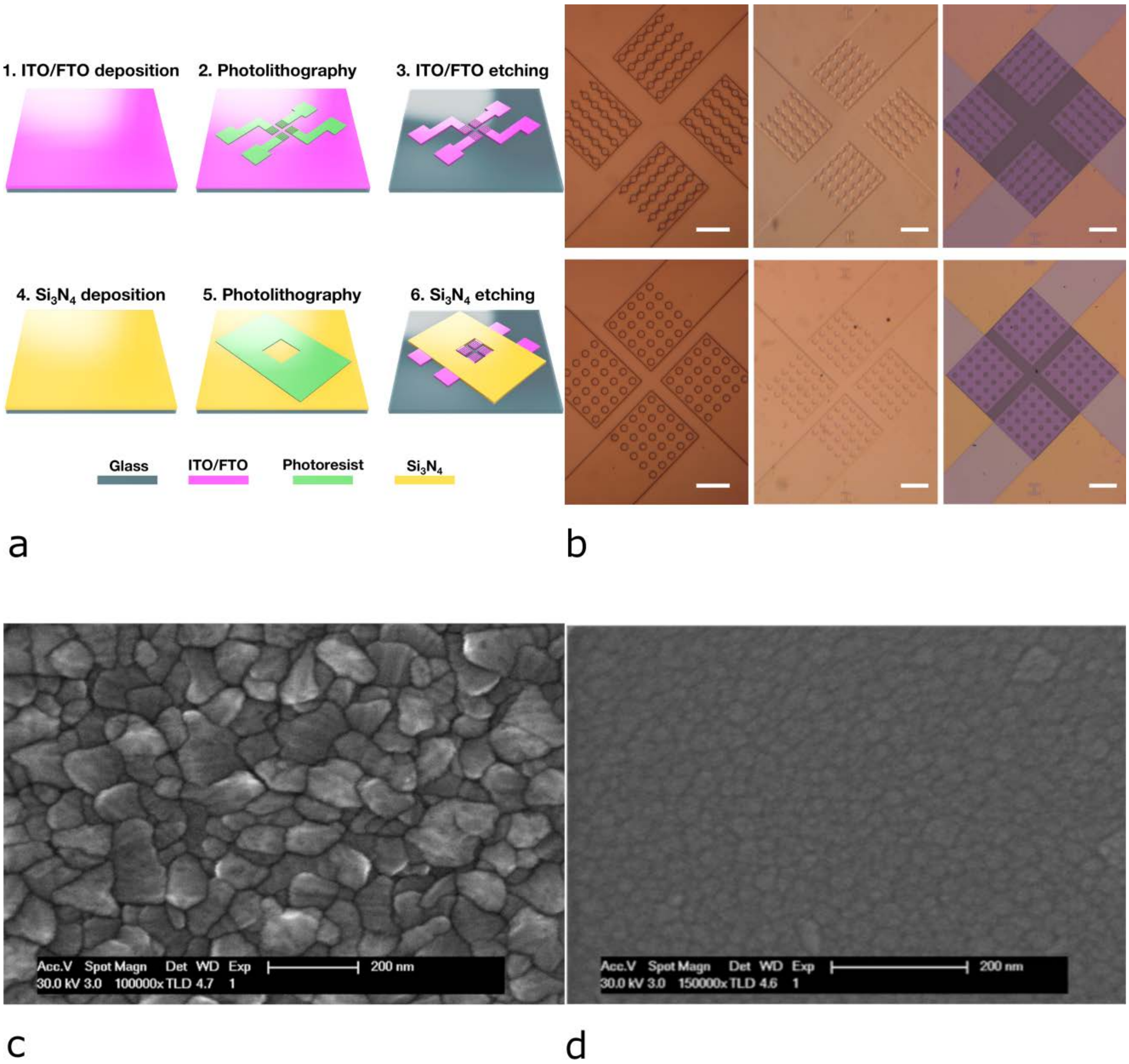

Figure S1. FTO and ITO electrodes used in the proposed opto-electrochemical approach. (**a**) Schematic overview of the electrode fabrication steps. (**b**) Optical microscopy images showing the central parts of typical ITO electrode arrays after steps 2, 3, and 6 (from left to right). (**c**) Scanning Electron Microscopy (SEM) image made on a FTO electrode. (**d**) SEM image made on an ITO electrode. Scale bars are 250 µm in the optical images and 200 nm in the SEM images.

Optical microscopy images of the central parts of typical electrode arrays are shown in **Fig. S1b**. As one can observe in **Fig. S1b**, while the lateral dimension of the electrodes was kept constant (500 µm × 500 µm), the inter-electrode spacing and the design of electrochemically inactive hole structure was modified. However, these parameters carry no significant importance in the current stage of our study. SEM images, revealing the higher surface roughness of the FTO electrodes as compared to the ITO electrodes, are shown in **Fig. S1c** and **S1d**. These SEM images confirm similar findings using Atomic Force Microscopy (**Fig. 1b**).

2. Electrochemical cell for opto-electrochemical measurements

To the best of our knowledge, there is no commercially available electrochemical cell that allows observing electrodes fabricated onto ~170 µm thick glass through the high magnification objectives (e.g. 63×) of an inverted microscope. Therefore, we constructed the electrochemical cell shown in **Fig. S2**. This electrochemical cell was built using a plastic microscope slide and it is compatible with common microscope stages. It holds the 2 cm × 2 cm × 170 µm glass slide carrying the metal oxide electrodes without hindering the access of the objectives of the inverted microscope to the electrode/solution interface. The cell can hold around 400 µL of solution. Due to this small volume of the cell, an Ag/AgCl wire was used as quasi-reference electrode. The stability of such a quasi-reference electrode was recently investigated and found satisfactory (~ 1 mV h^{-1}) [1].

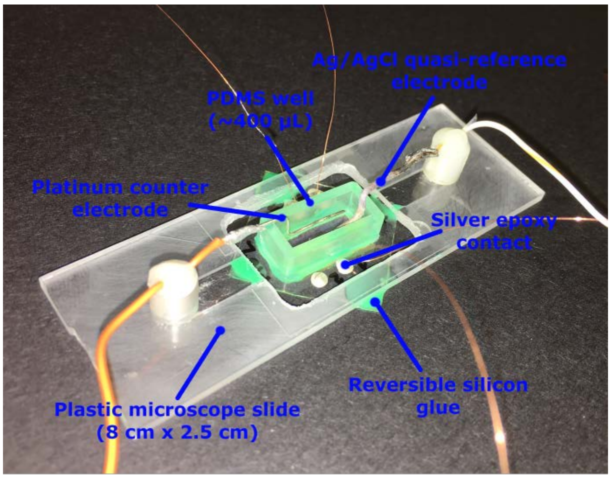

Figure S2. Electrochemical cell making our planar, optically transparent electrodes compatible with both electrochemical and optical methods based on an inverted microscope.

3. Reaction cascades providing the analytical useful signals

The current signals of the hydrogen peroxide and glucose biosensors investigated in the present work arise as a result of the biochemical and electrochemical processes schematically depicted in **Fig. S3a** and **S3b**. These reactions are presented into more details elsewhere[2,3]. Very important to note, the horseradish peroxidase (HRP) – hydrogen peroxide pair oxidizes the osmium complex-based redox polymer while the glucose oxidase (GOx) – glucose pair reduces the osmium complex-based redox polymer. Equally important to note, the processes schematically depicted in **Fig. S3a** and **S3b** cause also the BFRLM signal to change (because the refractive index of the redox polymer is different when the polymer is oxidized as compared to when it is reduced). However, the analyte concentration proportional optical signal is recorded with the potential of the electrode set to the OCP (i.e. in the absence of the electron transfer step in between the electrode and the redox polymer).

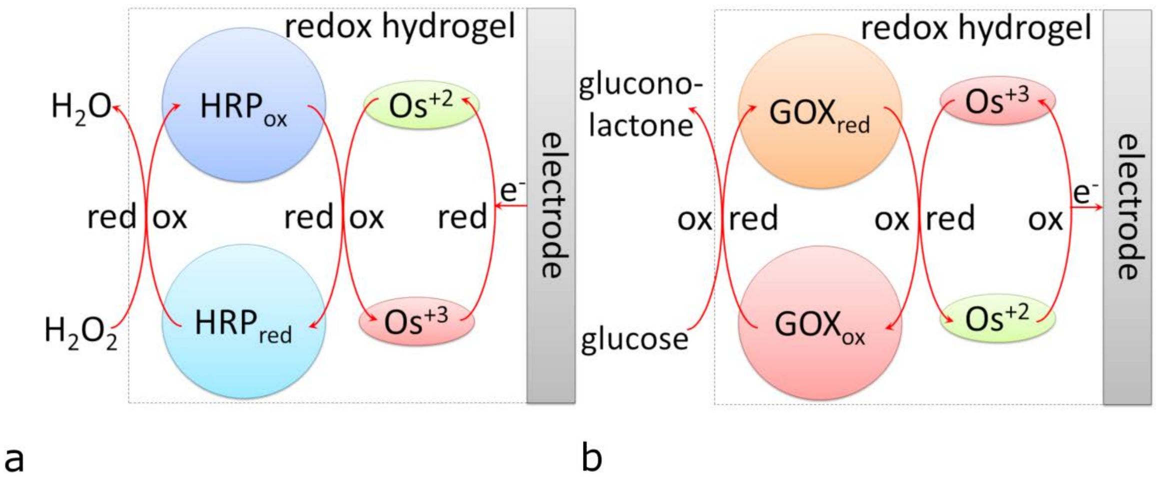

Figure S3. Redox cycles occurring in the used redox hydrogels when these are prepared with either HRP (**a**) or GOx (**b**) and are exposed to either hydrogen peroxide (**a**) or glucose (**b**).

4. Electrochemical detection of hydrogen peroxide using an ITO electrode modified with HRP-based redox hydrogel

The ability of redox hydrogel-modified metal oxide electrodes to detect hydrogen peroxide or glucose was always tested in combination with purely electrochemical methods. **Figure 2** of the main text shows the ability of a FTO electrode modified with a HRP-based redox hydrogel to detect hydrogen peroxide in combination with cyclic voltammetry and chronoamperometry. In this Supplementary Information, we show similar results obtained

with the remaining combinations of metal oxide electrodes (FTO and ITO) and redox hydrogels (with HRP and with GOx). **Figure S4** depicts the results obtained using an ITO electrode modified with a HRP-based redox hydrogel in combination with purely electrochemical methods (i.e. cyclic voltammetry and chronoamperometry).

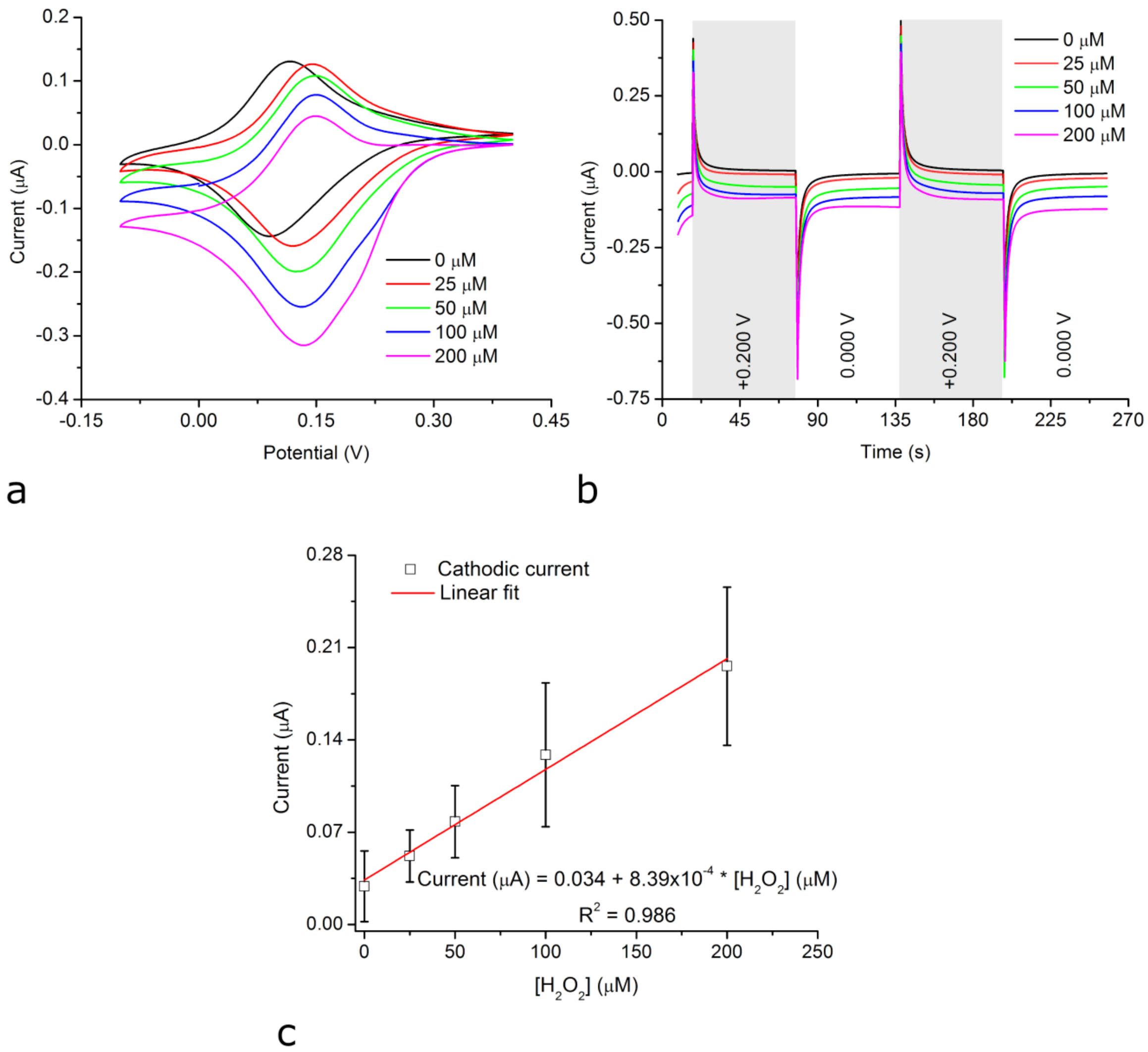

Figure S4. Electrochemical detection of hydrogen peroxide using an ITO electrode modified with HRP-based redox hydrogel. (**a**) Cyclic voltammograms of the modified ITO electrode recorded at a scan rate of 0.010 V s^{-1} in the presence of increasing concentrations of hydrogen peroxide. (**b**) Current signal of the modified ITO electrode when this is set to potentials oxidizing / reducing the redox polymer while the hydrogen peroxide concentration is increased stepwise. (**c**) Calibration curve obtained using currents recorded at the end of the second cathodic pulse.

As one can see in **Fig. S4a**, the cyclic voltammograms of the ITO electrode modified with HRP-based redox hydrogel display two current peaks corresponding to the oxidation and the reduction of the redox polymer just as the voltammograms of the similar FTO electrode exhibited in **Fig. 2b**. The peak potential difference characterizing the HRP-based redox hydrogel on the ITO electrode is significantly smaller than the peak potential difference of same hydrogel on the FTO electrode (0.027 V *vs.* 0.091 V). In the presence of hydrogen peroxide, the current corresponding to the reduction of the redox polymer increases and the current corresponding to the oxidation of the redox polymer decreases. This behaviour of the ITO electrode modified with HRP-based redox hydrogel is in perfect line with the reaction cascade depicted in **Fig. S3a** according to which the HRP – hydrogen peroxide pair oxidizes the redox polymer of the hydrogel. During the chronoamperometric experiments, the potential of the ITO electrode modified with HRP-based redox hydrogel was set to either +0.200 V (to oxidize the redox polymer) or to 0.000 V (to reduce the redox polymer) and the concentration of the hydrogen peroxide in the solution bathing the electrode was increased stepwise from 0 to 200 µM. The resulting (anodic and cathodic) currents were found to depend on the hydrogen peroxide concentration (**Fig. S4b**). The currents, recorded at the end of the second cathodic pulse (i.e., at t = 256 s), allowed building the calibration curve shown in **Fig. S4c** (average of 4 experiments with 4 different hydrogel-modified ITO electrodes). The sensitivity of the hydrogen peroxide biosensors built on ITO, calculated as the slope of the linear range of the calibration curve, is with ~ 29% higher than the similarly calculated sensitivity of the hydrogen peroxide biosensor built on FTO (8.39 10^{-4} µA $µM^{-1}$ *vs.* 5.99 10^{-4} µA $µM^{-1}$). As detailed in the main text, this higher sensitivity of the ITO-based biosensors shows up not only when using purely electrochemical methods, but also in our opto-electrochemical approach. The coefficient of variation characterizing the sensitivity was 27% for the electrochemical sensors for hydrogen peroxide detection made with ITO and 15% for similar sensors made with FTO. The detection limit and the limit of quantitation were found to be 90 µM and 310 µM, respectively. These limits are larger than those obtained for similar sensors made with FTO (15 µM and 70 µM, respectively).

5. Electrochemical detection of glucose using a FTO electrode modified with GOx-based redox hydrogel

Figure S5 exhibits results obtained with a FTO electrode modified with GOx-based redox hydrogel in combination with purely electrochemical methods (i.e., cyclic voltammetry and

chronoamperometry). The cyclic voltammograms of the FTO electrode modified with GOx-based redox hydrogel display two current peaks corresponding to the oxidation and the reduction of the redox polymer, at +0.238 V and +0.192 V, respectively (**Fig. S5a**). In the presence of glucose, the current corresponding to the oxidation of the redox polymer increased and the current corresponding to the reduction of the redox polymer decreased.

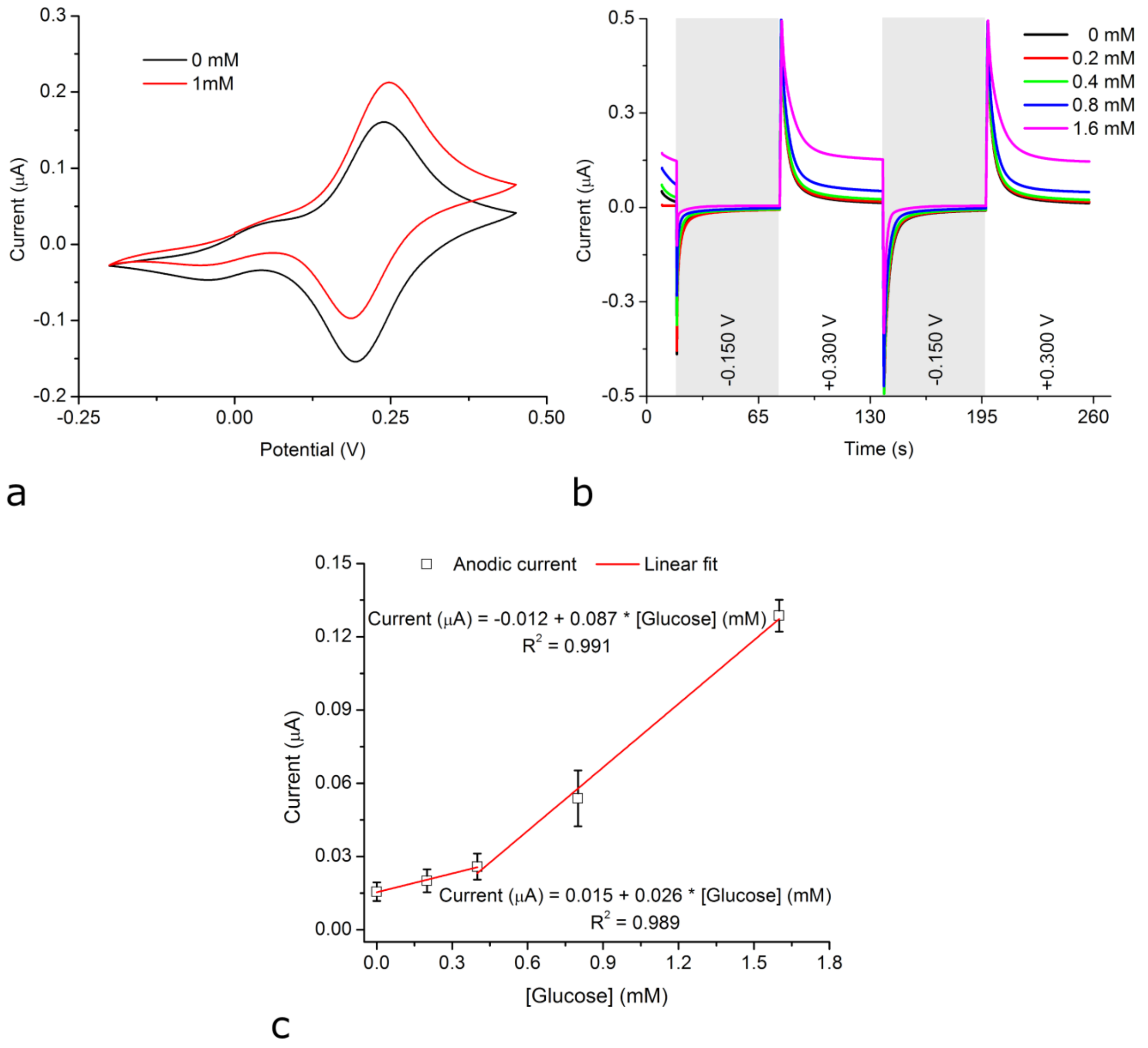

Figure S5. Electrochemical detection of glucose using a FTO electrode modified with GOx-based redox hydrogel. (**a**) Cyclic voltammograms of the modified FTO electrode recorded at a scan rate of 0.010 V s^{-1} in the absence and the presence of glucose. (**b**) The current signal of the modified FTO electrode when this is set to potentials oxidizing / reducing the redox polymer while the glucose concentration is increased stepwise. (**c**) Calibration curve obtained using currents recorded at the end of the second anodic pulse.

These results of the cyclic voltammetry study are in perfect line with the reaction cascade depicted in **Fig. S3b**, according to which the GOx – glucose pair reduces the redox polymer and the electrode oxidizes the redox polymer. During the chronoamperometric experiments, the potential of the FTO electrode modified with GOx-based redox hydrogel was set to either +0.300 V (to oxidize the redox polymer) or to -0.150 V (to reduce the redox polymer) and the concentration of the glucose in the solution bathing the electrode was increased stepwise from 0 to 1.6 mM. The resulting currents were observed to depend on the glucose concentration (**Fig. S5b**). The anodic currents, recorded at the end of the second anodic pulse (i.e. at t = 256 s), allowed building the calibration curve shown in **Fig. S5c** (average of 3 experiments with 3 different hydrogel-modified FTO electrodes). This calibration curve presents two regions: a first region characterized by a smaller increase of the current signal with the glucose concentration (up to 0.4 mM glucose), and a second region characterized by a somewhat larger increase of the current with the glucose concentration (from 0.4 to 1.6 mM). Interestingly enough, the calibration curve characterizing such sensors presented the same two regions even when built using the optical signals of a single region of interest (ROI) defined on the sensor/solution interface (instead of the current signals of the whole sensor – solution interface, see **Fig. S5c** *vs.* **Fig. 4c**). This sustains the robustness of our opto-electrochemical approach. The coefficient of variation characterizing the sensitivity of electrochemical sensors for glucose detection made with FTO was as small as 4%. The detection limit and the limit of quantitation were found to be 450 µM and 760 µM, respectively.

6. Electrochemical detection of glucose using an ITO electrode modified with GOx-based redox hydrogel

Figure S6 shows the results obtained with an ITO electrode modified with a GOx-based redox hydrogel in combination with cyclic voltammetry and chronoamperometry. The cyclic voltammograms of the ITO electrode modified with GOx-based redox hydrogel display two current peaks (**Fig. S6a**). The peak at +0.244 V is due to the oxidation of the redox polymer found in the hydrogel and the current peak at +0.190 V is due to the reduction of the same polymer. In the presence of glucose, the current corresponding to the oxidation of the redox polymer increased while the current due to the reduction of the redox polymer decreased. These results of the cyclic voltammetry study are in agreement with the reaction cascade depicted in **Fig. S3b**. During the chronoamperometric experiments, the potential of the FTO electrode modified with GOx-based redox hydrogel was set to either +0.300 V (to oxidize the

redox polymer) or to -0.150 V (to reduce the redox polymer) and the concentration of the glucose in the solution bathing the electrode was stepwise increased from 0 to 1.6 mM. The resulting currents were observed to depend on the glucose concentration (**Fig. S6b**). The currents recorded at the end of the second anodic pulse allowed building the calibration curve shown in **Fig. S6c** (average of 9 experiments with 9 different hydrogel-modified ITO electrodes).

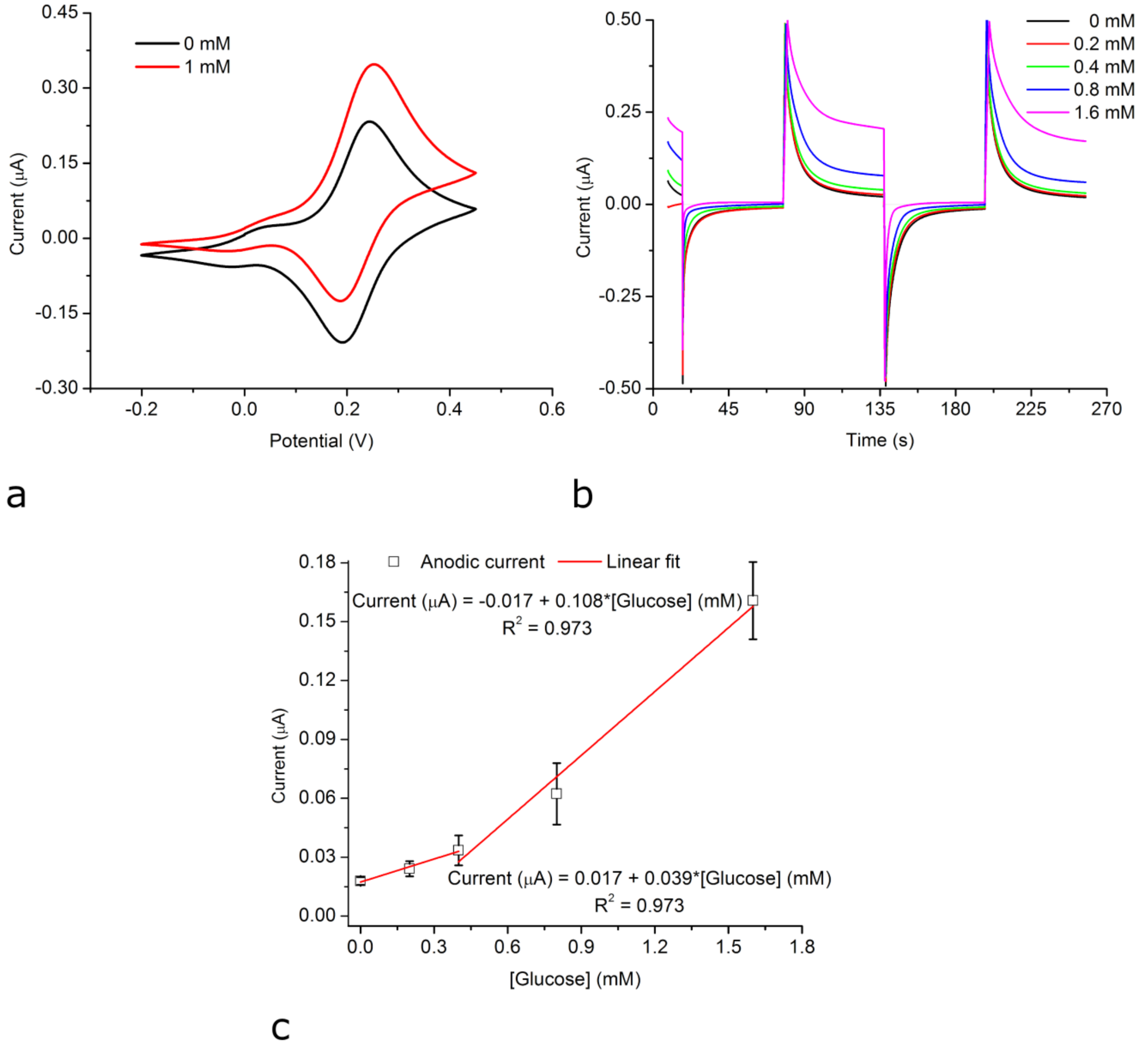

Figure S6. Electrochemical detection of glucose using an ITO electrode modified with GOx-based redox hydrogel. (**a**) Cyclic voltammograms of the modified ITO electrode recorded at a scan rate of 0.010 V s^{-1} in the absence and the presence of glucose. (**b**) Current signal of the modified FTO electrode when this is set to potentials oxidizing / reducing the redox polymer while the glucose concentration is increased stepwise. (**c**) Calibration curve obtained using currents recorded at the end of the second anodic pulse.

The sensitivity of this glucose biosensor built on ITO (calculated as the slope of the linear range of the calibration curve observed from 0.4 mM to 1.6 mM glucose) was ~ 19% higher than that of similar biosensors built on FTO (0.108 µA mM^{-1} *vs.* 0.087 µA mM^{-1}). This confirms that ITO electrodes allow building better biosensors than FTO electrodes for both hydrogen peroxide and glucose detection. Moreover, this behaviour is maintained both when the biosensors are interrogated with purely electrochemical methods (as described in this Supplementary Information) and when they are interrogated with our opto-electrochemical approach (as described in the main text). The coefficient of variation characterizing the sensitivity of electrochemical sensors for glucose detection made with ITO was 12% (*vs.* 4% for similar sensors made using FTO). The detection limit and the limit of quantitation were found to be 170 µM and 510 µM, respectively. These limits are smaller than those obtained for similar sensors made with FTO (450 µM and 760 µM, respectively).

7. Opto-electrochemical detection of hydrogen peroxide using an ITO electrode modified with HRP-based redox hydrogel

Figure 3 of the main text shows the ability of an FTO electrode modified with HRP-based redox hydrogel to detect hydrogen peroxide with a spatial resolution of 18 µm × 18 µm when interrogated with both chronoamperometry and BFRLM. In this section of the Supplementary Information, we show similar results obtained with an ITO electrode modified with a HRP-based redox hydrogel. This electrode was investigated using BFRLM and a higher magnification objective (63× instead of 40×). **Figure S7a** shows a BFRLM image with 48 ROIs of the ITO electrode modified with HRP-based redox hydrogel. **Figure S7b** depicts how the mean intensity of one of the ROIs (found on ITO) changes when the potential of the electrode is set twice to +0.200 V (to oxidize the redox polymer), twice to -0.000 V (to reduce the redox polymer), and then left at open circuit potential (OCP; starting with t = 257 s) both in the absence and in the presence of different concentrations of hydrogen peroxide. The results obtained with the ITO electrode and a 63× magnification objective are qualitatively similar to those achieved with the FTO electrode and the 40× magnification objective (see **Fig. S7b** *vs.* **Fig. 3b**). However, the evolution of the corrected mean intensity while changing applied potentials and hydrogen peroxide concentrations is somewhat noisier due to the smaller physical dimensions of the ROIs (12 µm × 12 µm *vs.* 18 µm × 18 µm). The sensitivity of the ROIs to hydrogen peroxide was nevertheless preserved in spite of the noisier optical signals.

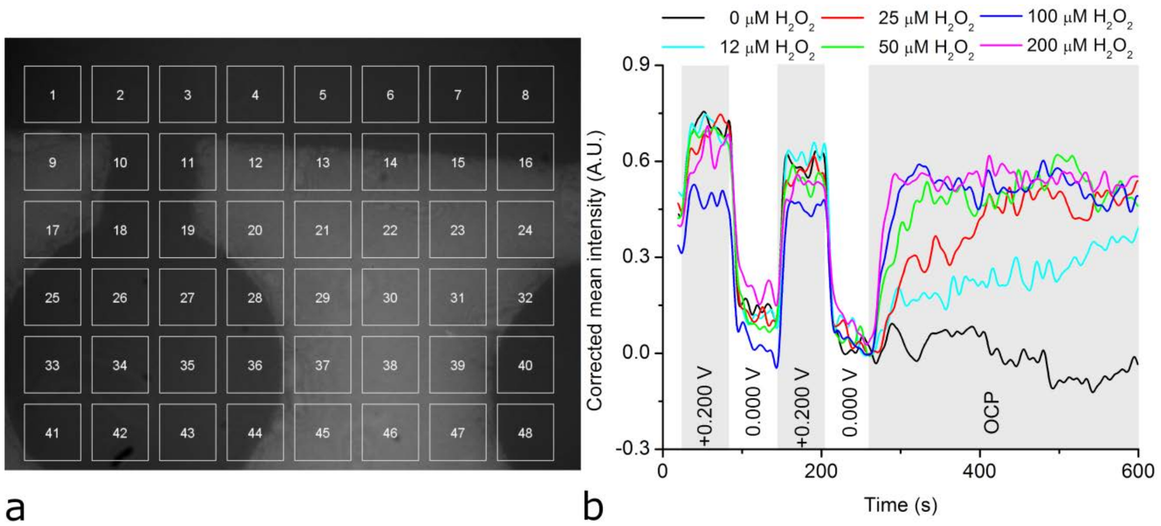

Figure S7. Opto-electrochemical detection of hydrogen peroxide using an ITO electrode modified with HRP-based redox hydrogel. (a) BFRLM image with 48 ROIs with dimensions of 12 µm × 12 µm each of the ITO electrode modified with HRP-based redox hydrogel. **(b)** Evolution of the optical signal for ROI no. 24 when the ITO electrode modified with HRP-based redox hydrogel was first polarized to potentials oxidizing / reducing the redox polymer and then left at OCP while the hydrogen peroxide concentration was increased stepwise.

8. Opto-electrochemical detection of glucose using an ITO electrode modified with GOx-based redox hydrogel

Figure 4 of the main text shows the ability of an FTO electrode modified with GOx-based redox hydrogel to detect glucose with a spatial resolution of 18 µm × 18 µm when interrogated with both chronoamperometry and BFRLM. In this section of the Supplementary Information, we show similar results obtained with an ITO electrode modified with a GOx-based redox hydrogel. **Figure S8a** shows a BFRLM image with 48 ROIs of the ITO electrode modified with GOx-based redox hydrogel. **Figure S8b** depicts how the mean intensity of one of the ROIs (found on ITO) changes when the potential of the electrode is set twice to +0.300 V (to oxidize the redox polymer), twice to -0.150 V (to reduce the redox polymer), and then left at OCP both in the absence and in the presence of different concentrations of glucose. The results obtained with the ITO electrode are qualitatively similar to those achieved with the FTO electrode (see **Fig. S8b** *vs.* **Fig. 4b**).

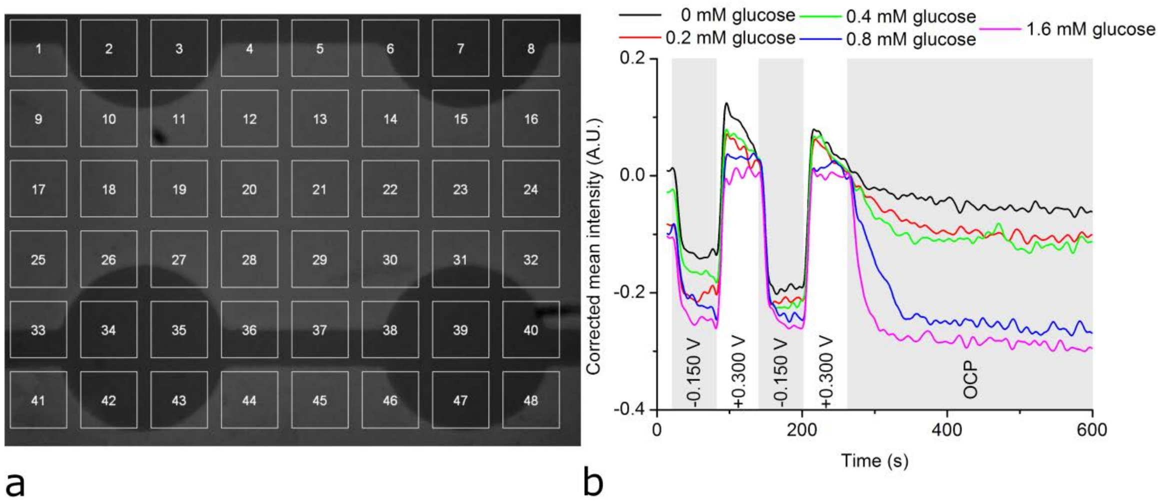

Figure S8. Opto-electrochemical detection of glucose using an ITO electrode modified with GOx-based redox hydrogel. (a) BFRLM image with 48 ROIs with dimensions of 18 µm × 18 µm each of the ITO electrode modified with GOx-based redox hydrogel. **(b)** Evolution of the optical signal for ROI no. 12 when the ITO electrode modified with GOx-based redox hydrogel was first polarized to potentials oxidizing / reducing the redox polymer and then left at OCP while the glucose concentration was increased stepwise.

9. Selectivity of the developed opto-electrochemical sensors

Redox hydrogel-based electrochemical biosensors are most often characterized by good selectivity because they work with low applied potentials[4]. As a result, they were already used in complex environments such as brain tissue[5,6]. Moreover, due to its excellent analytical performances, a redox hydrogel-based glucose sensor was also integrated into a commercial glucose meter[7]. However, the selectivity of redox hydrogel-based electrochemical biosensors is often boosted by using additional, permselective polymer layers deposited on top of the redox hydrogel. We investigated the selectivity of the developed opto-electrochemical sensors in their current form, with no additional permselective polymer layers. The selectivity of opto-electrochemical sensors built on ITO was investigated by challenging the hydrogen peroxide sensor with glucose and the glucose sensor with hydrogen peroxide. The results of these selectivity tests are shown in **Fig. S9**.

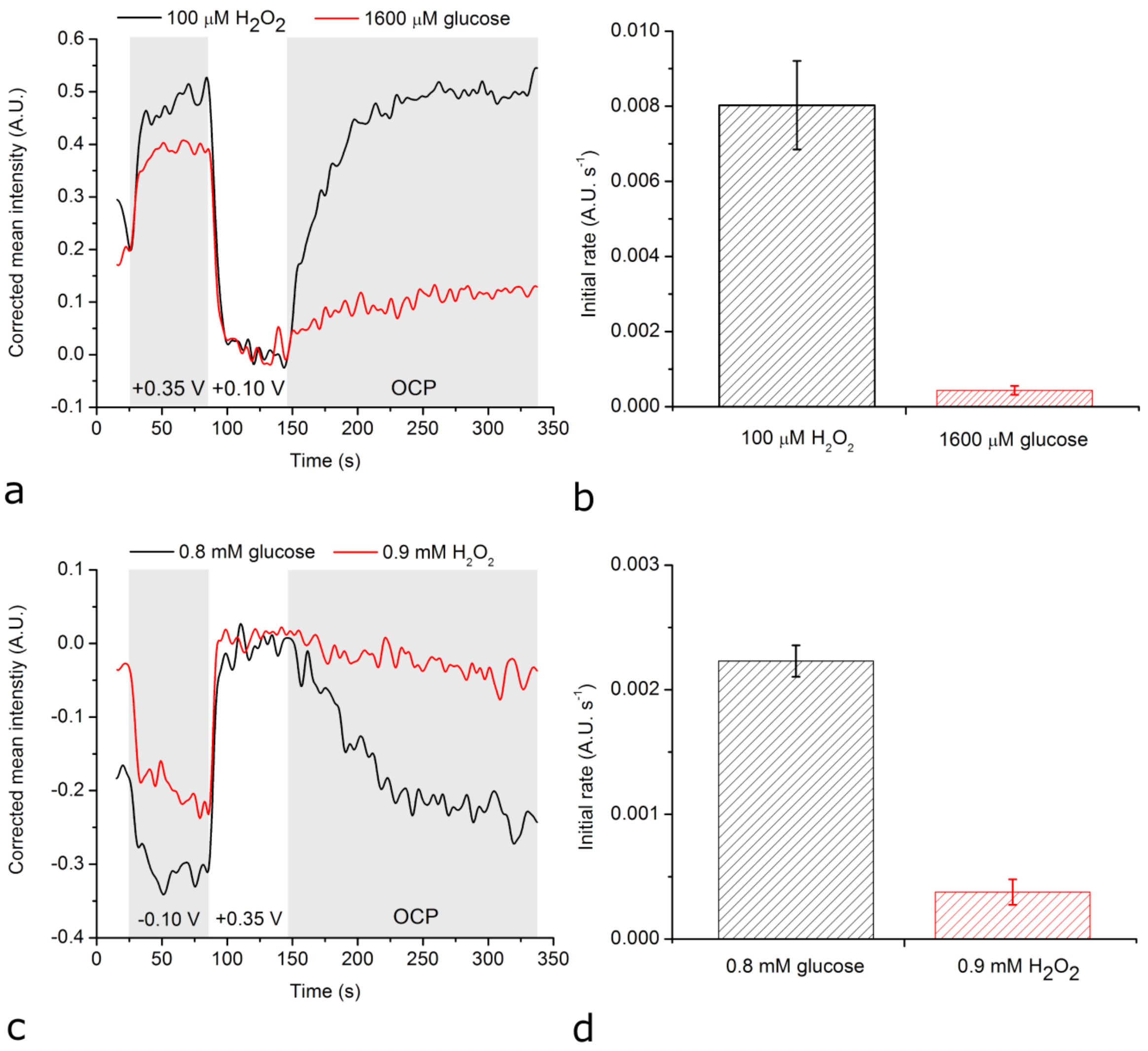

Figure S9. Selectivity of opto-electrochemical sensors developed using ITO and redox hydrogels. (a) Evolution of the optical signal of a single ROI (18 µm × 18 µm) when the ITO electrode modified with HRP-based redox hydrogel was first polarized to potentials oxidizing / reducing the redox polymer and then left at OCP in the presence of either 100 µM hydrogen peroxide or 1600 µM glucose. **(b)** Initial rates observed optically with the ITO electrode modified with HRP-based redox hydrogel (average values obtained from at least 10 ROIs). **(c)** evolution of the optical signal of a single ROI (18 µm × 18 µm) when the ITO electrode modified with GOx-based redox hydrogel was first polarized to potentials reducing / oxidizing the redox polymer and then left at OCP in the presence of either 0.8 mM glucose or 0.9 mM hydrogen peroxide. **(d)** Initial rates observed optically with the ITO electrode modified with GOx-based redox hydrogel (average values obtained from at least 10 ROIs).

As one can observe in **Fig. S9a** and **S9b**, 1.6 mM glucose produced a signal corresponding to only ~ 5 µM hydrogen peroxide on the opto-electrochemical hydrogen peroxide sensor. In the same time, 0.9 mM hydrogen peroxide (a very high concentration taking into account the

envisaged applications, e.g., measurements at cellular level) produced a signal corresponding to ~ 140 μM glucose on the opto-electrochemical glucose sensor (**Fig. S9c** and **S9d**). Both signals produced by the non-target compounds correspond to analyte concentrations smaller than the detection limits of the opto-electrochemical sensors (which were calculated to be 20 μM and 320 μM for the opto-electrochemical hydrogen peroxide sensor and the opto-electrochemical glucose sensor, respectively). Both signals can be further decreased by using relatively simple methods described in the literature for improving the selectivity of redox hydrogel-based biosensors (e.g., by using an additional Nafion layer deposited on top of the redox hydrogel[5,6]).

10. References

1. Bentley, C. L., Perry, D. & Unwin, P. R. Stability and placement of Ag/AgCl quasi-reference counter electrodes in confined electrochemical cells. *Anal. Chem.* **90**, 7700–7707 (2018).
2. Gregg, B. A. & Heller, Adam. Cross-linked redox gels containing glucose oxidase for amperometric biosensor applications. *Anal Chem* **62**, 258–263 (1990).
3. Vreeke, M. S. & Heller, A. Hydrogen peroxide electrodes based on electrical connection of redox centers of various peroxidases to electrodes through a three-dimensional electron-relaying polymer network. in *Diagnostic Biosensor Polymers* (eds. Usmani, A. M. & Akmal, N.) **556**, 180–192 (American Chemical Society, 1994).
4. Ohara, T. J., Rajagopalan, Ravi. & Heller, Adam. 'Wired' Enzyme Electrodes for Amperometric Determination of Glucose or Lactate in the Presence of Interfering Substances. *Anal. Chem.* **66**, 2451–2457 (1994).
5. Kulagina, N. V., Shankar, L. & Michael, A. C. Monitoring Glutamate and Ascorbate in the Extracellular Space of Brain Tissue with Electrochemical Microsensors. *Anal. Chem.* **71**, 5093–5100 (1999).
6. Kulagina, N. V. & Michael, A. C. Monitoring Hydrogen Peroxide in the Extracellular Space of the Brain with Amperometric Microsensors. *Anal. Chem.* **75**, 4875–4881 (2003).
7. Heller, A. & Feldman, B. Electrochemistry in Diabetes Management. *Acc. Chem. Res.* **43**, 963–973 (2010).
